# Analgesic Effects of Bee Venom Derived Phospholipase A_2_ in a Mouse Model of Oxaliplatin-Induced Neuropathic Pain

**DOI:** 10.3390/toxins7072422

**Published:** 2015-06-29

**Authors:** Dongxing Li, Younju Lee, Woojin Kim, Kyungjin Lee, Hyunsu Bae, Sun Kwang Kim

**Affiliations:** 1 Department of Physiology, College of Korean Medicine, Kyung Hee University, 26 Kyungheedae-ro, Dongdamoon-gu, Seoul 130-701, Korea; E-Mails: leedongxing@naver.com (D.L.); eyounju@naver.com (Y.L.); hbae@khu.ac.kr (H.B.); 2 Department of East-West Medicine, Graduate School, Kyung Hee University, 26 Kyungheedae-ro, Dongdamoon-gu, Seoul 130-701, Korea; E-Mail: thasnow@gmail.com; 3 Department of Herbology, College of Korean Medicine, Kyung Hee University, 26 Kyungheedae-ro, Dongdamoon-gu, Seoul 130-701, Korea; E-Mail: niceday@khu.ac.kr

**Keywords:** phospholipase A_2_, Bee Venom, oxaliplatin, neuropathic pain, noradrenergic

## Abstract

A single infusion of oxaliplatin, which is widely used to treat metastatic colorectal cancer, induces specific sensory neurotoxicity signs that are triggered or aggravated when exposed to cold or mechanical stimuli. Bee Venom (BV) has been traditionally used in Korea to treat various pain symptoms. Our recent study demonstrated that BV alleviates oxaliplatin-induced cold allodynia in rats, via noradrenergic and serotonergic analgesic pathways. In this study, we have further investigated whether BV derived phospholipase A_2_ (bvPLA_2_) attenuates oxaliplatin-induced cold and mechanical allodynia in mice and its mechanism. The behavioral signs of cold and mechanical allodynia were evaluated by acetone and a von Frey hair test on the hind paw, respectively. The significant allodynia signs were observed from one day after an oxaliplatin injection (6 mg/kg, i.p.). Daily administration of bvPLA_2_ (0.2 mg/kg, i.p.) for five consecutive days markedly attenuated cold and mechanical allodynia, which was more potent than the effect of BV (1 mg/kg, i.p.). The depletion of noradrenaline by an injection of *N*-(2-chloroethyl)-*N*-ethyl-2-bromobenzylamine hydrochloride (DSP4, 50 mg/kg, i.p.) blocked the analgesic effect of bvPLA_2_, whereas the depletion of serotonin by injecting DL-*p*-chlorophenylalanine (PCPA, 150 mg/kg, i.p.) for three successive days did not. Furthermore, idazoxan (α2-adrenegic receptor antagonist, 1 mg/kg, i.p.) completely blocked bvPLA_2_-induced anti-allodynic action, whereas prazosin (α1-adrenegic antagonist, 10 mg/kg, i.p.) did not. These results suggest that bvPLA_2_ treatment strongly alleviates oxaliplatin-induced acute cold and mechanical allodynia in mice through the activation of the noradrenergic system, via α2-adrenegic receptors, but not via the serotonergic system.

## 1. Introduction

Oxaliplatin is an effective platinum derivative, which is widely used in the treatment of colorectal carcinoma [1,2], but causes neurotoxicity predominantly within the peripheral nervous system [3,4]. Two different types of oxaliplatin-induced peripheral neuropathy have been described hitherto, *i.e.*, cold and mechanical hypersensitivity [5,6]. However, effective treatment for oxaliplatin-induced cold and mechanical hypersensitivity still remains to be elucidated. Hence, it is required to discover therapeutic options for the management of oxaliplatin-induced neuropathic pain.

Bee Venom (BV) has been traditionally used in Korea to relieve pain and to treat chronic inflammatory diseases [7,8,9,10,11,12]. Previous studies have demonstrated that the analgesic effects of BV in various pain models are mediated mainly by activation of α2-adrenergic and/or serotonergic receptors [12,13,14,15,16]. Rho and his colleagues have reported that subcutaneous injections of BV attenuated heat hyperalgesia and cold and mechanical allodynia in the rats with nerve injury-induced neuropathic pain through the activation of the endogenous noradrenergic system [17,18]. In a rat model of oxaliplatin-induced neuropathic pain, we found that the anti-allodynic effect of BV is at least partially mediated by the noradrenergic and serotonergic system, but not by the opioid system [9,16].

Phospholipase A_2_ from BV (bvPLA_2_), a prototypic group III enzyme that hydrolyzes fatty acids in membrane phospholipids, is one of the major active components of BV [19,20]. Several studies have shown that this bvPLA_2_ prevents neuronal cell death and spinal cord injury [21,22]. Our previous study demonstrated that BV mitigates cisplatin-induced nephrotoxicity [23] and found that bvPLA_2_ can reduce such nephrotoxicity more potently than BV [24]. However, the effect of PLA_2_ on oxaliplatin-induced neuropathic pain and its mechanism have not been studied yet.

The aim of this study was to evaluate and compare the analgesic effect of BV and bvPLA_2_ on oxaliplatin-induced cold and mechanical allodynia in mice. In addition, we examined whether the anti-allodynic effect of bvPLA_2_ is mediated by the serotonergic or noradrenergic pain inhibitory system.

## 2. Results

### 2.1. Effects of BV and bvPLA_2_ on Oxaliplatin-Induced Cold and Mechanical Hypersensitivity

**Figure 1:**
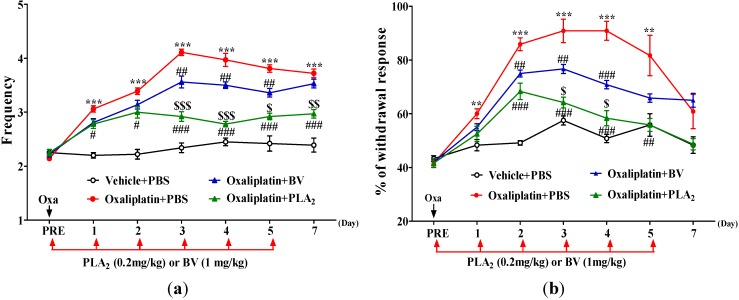
Effects of BV and bvPLA_2_ on oxaliplatin-induced cold and mechanical allodynia in mice. The behavioral tests for cold (**a**) and mechanical (**b**) allodynia were performed before (PRE) and after the administration of oxaliplatin (6 mg/kg, i.p.). Vehicle (5% glucose) + PBS, Oxaliplatin + PBS, Oxaliplatin + BV, and Oxaliplatin + bvPLA_2_ groups received daily injection of PBS, BV (1 mg/kg, i.p.) or bvPLA_2_ (0.2 mg/kg, i.p.) for five days after an oxaliplatin or vehicle administration. Results are expressed as mean ± SEM; *n* = 6 mice/group; The data was analyzed with one-way ANOVA followed by the Tukey’s multiple comparison test. ** *p* < 0.01, *** *p* < 0.001, *vs.* Vehicle + PBS; ^#^ *p* < 0.05, ^##^ *p* < 0.01, ^###^ *p* < 0.001 *vs.* Oxaliplatin + PBS; ^$^ p < 0.05, ^$$^ *p* < 0.01, ^$$$^ *p* < 0.001, *vs.* Oxaliplatin + BV.

First, we investigated the effects of a single administration of oxaliplatin (6 mg/kg, i.p.) on behavioral sensitivity to cold and mechanical stimuli in mice (Figure 1). The administration significantly increased the frequency of licking and shaking of the hind paw in response to cold acetone stimuli. A significant cold allodynia was observed at day 1, peaked at day 3 and lasted for at least seven days after the oxaliplatin administration, compared to the vehicle group (Figure 1a). Similarly, an administration of oxaliplatin significantly increased the withdrawal responses of the hind paw to von Frey filament applications (as expressed % of withdrawal response) at day 1, peaked at day 3–4 and maintained up to day 5 (Figure 1b).

Daily BV treatment (1 mg/kg, i.p.) for five consecutive days significantly reduced the cold allodynia from three days after the oxaliplatin administration and such analgesic effect last up to day 5. In addition, bvPLA_2_ treatment (0.2 mg/kg, i.p.) significantly attenuated the cold allodynia from day 1 after the oxaliplatin injection and such effect endured at least for the following six days (Figure 1a). BV treatment also significantly attenuated mechanical allodynia from day 2 after the oxaliplatin injection and this BV analgesia was continued up to day 4. Moreover, bvPLA_2_ treatment showed a significant reduction in mechanical allodynia from day 2 after the oxaliplatin injection and such effect lasted up to day 5 (Figure 1b). The relieving effects of bvPLA_2_ on oxaliplatin-induced cold and mechanical allodynia were significantly more potent than those of BV (Figure 1).

### 2.2. Effects of BvPLA_2_ on Oxaliplatin-Induced Cold and Mechanical Allodynia in Serotonin Depleted Mice

**Figure 2:**
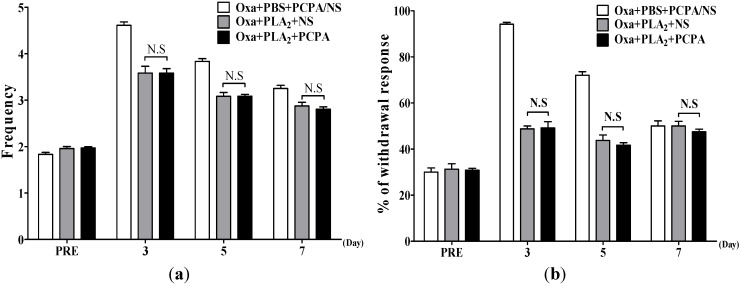
Effects of bvPLA_2_ on oxaliplatin-induced cold and mechanical allodynia in serotonin depleted mice. The behavioral tests for cold (**a**) and mechanical (**b**) allodynia were performed before and after an administration of oxaliplatin (Oxa, 6 mg/kg, i.p.). Serotonin was depleted by daily injections of PCPA (150 mg/kg, i.p.) for three consecutive days prior to an oxaliplatin administration. Oxa + PBS + PCPA/NS, *n* = 10; Other groups, *n* = 6 mice/group; Results are expressed as mean ± SEM; N.S, no significance (*p* > 0.05), The data were analyzed with one-way ANOVA followed by the Tukey’s multiple comparison test.

We investigated the effects of bvPLA_2_ on oxaliplatin-induced cold and mechanical allodynia in serotonin depleted mice by injecting DL-*p*-chlorophenylalanine (PCPA, 150 mg/kg, i.p.) for three successive days [25,26]. PCPA pretreatment itself did not affect the behavioral signs of cold and mechanical allodynia induced by oxaliplatin (*p* > 0.05, Oxaliplatin + PBS + PCPA [*n* = 6] *vs.* Oxaliplatin + PBS + NS [*n* = 4], Cold (frequency): 1.83 ± 0.04 *vs.* 1.92 ± 0.05 at 0d, 4.61 ± 0.07 *vs.* 4.67 ± 0.07 at 3d, 3.83 ± 0.06 *vs.* 3.79 ± 0.08 at 5d, 3.25 ± 0.07 *vs.* 3.04 ± 0.10 at 7d; Mechanical (%): 30.00 ± 1.83 *vs.* 30.00 ± 0.00 at 0d, 94.17 ± 0.83 *vs.* 93.75 ± 1.25 at 3d, 72.03 ± 1.54 *vs.* 68.75 ± 1.25 at 5d, 50 ± 2.24 *vs.* 51.25 ± 1.25 at 7d). Thus, we pooled the data from the two groups as a control group (Oxaliplatin + PBS + PCPA/NS, Figure 2). Compared to this control group, bvPLA_2_ treatment in mice without serotonin depletion (Oxaliplatin + PLA_2_ + NS group) significantly attenuated the cold and mechanical hypersensitivity (*p* < 0.01 at days 3 and 5). Such anti-allodynic effects of bvPLA_2_ were not blocked by PCPA pretreatment (Figure 2), indicating that the serotonergic mechanism is not involved in the analgesic effect of bvPLA_2_ on oxaliplatin-induced neuropathic pain.

### 2.3. Noradrenergic Mechanism of the Anti-Allodynic Effects of BvPLA_2_ in Oxaliplatin-Administered Mice

**Figure 3:**
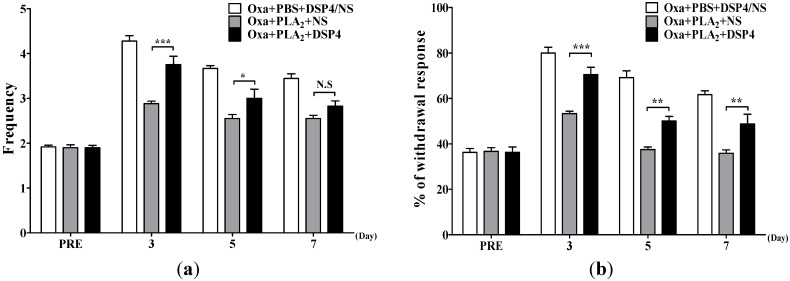
Effects of bvPLA_2_ on oxaliplatin-induced cold and mechanical allodynia in noradrenaline depleted mice. The behavioral tests for cold (**a**) and mechanical (**b**) allodynia were performed before and after an administration of oxaliplatin (6 mg/kg, i.p.). Noradrenaline was depleted by an injection of DSP4 (50 mg/kg, i.p.) at a day before an oxaliplatin administration. Since DSP4 pretreatment itself did not affect the cold and mechanical allodynia signs induced by oxaliplatin (*p* > 0.05, Oxa + PBS + DSP4 [*n* = 6] *vs.* Oxa + PBS + NS [*n* = 6]), we pooled the data from the two groups as a control (Oxa + PBS + DSP4/NS, n = *12*). Other groups, *n* = 6 mice/group; Results are expressed as mean ± SEM; N.S, no significance (*p* > 0.05), * *p* < 0.05, ** *p* < 0.01, *** *p* < 0.001, The data was analyzed with one-way ANOVA followed by the Tukey’s multiple comparison test.

We evaluated the effects of bvPLA_2_ on oxaliplatin-induced allodynia in noradrenaline depleted mice by a pretreatment of DSP4 [27]. The anti-allodynic effects of bvPLA_2_ (Oxa + PLA_2_ + NS group, *p* < 0.01 *vs.* control Oxa + PBS + DSP4/NS group at days 3, 5 and 7) were significantly blocked by DSP4 pretreatment (Figure 3), unlike the aforementioned PCPA pretreatment. These results suggest that activation of the noradrenergic pain inhibitory pathway at least partially mediates the bvPLA_2_-induced anti-allodynic action in oxaliplatin-administered mice.

**Figure 4:**
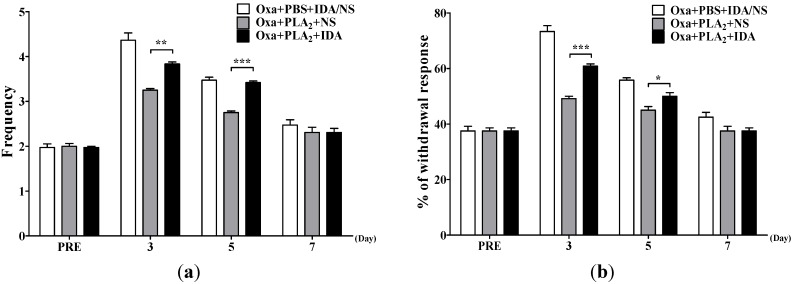
Effect of α2-adrenergic receptor antagonist, idazoxan, on bvPLA_2_-induced anti-allodynic action in oxaliplatin-administered mice. The behavioral tests for cold (**a**) and mechanical (**b**) allodynia were performed before and after an administration of oxaliplatin (6 mg/kg, i.p.). Idazoxan (IDA, 1 mg/kg, i.p.) was administered 30 min prior to bvPLA_2_ injection. Since IDA pretreatment itself did not affect the cold and mechanical allodynia signs induced by oxaliplatin (*p* > 0.05, Oxa + PBS + IDA [*n* = 6] *vs.* Oxa + PBS + NS [*n* = 6]), we pooled the data from the two groups as a control (Oxa + PBS + IDA/NS, *n* = 12). Other groups, *n* = 6 mice/group; Results are expressed as mean ± SEM; * *p* < 0.05, ** *p* < 0.01, *** *p* < 0.001, The data was analyzed with one-way ANOVA followed by the Tukey’s multiple comparison test.

To identify which adrenergic receptor subtype mediates the analgesic effects of bvPLA_2_ on oxaliplatin-induced neuropathic pain in mice, we examined the effect of prazosin (PRA, α1-adrenergic receptor antagonist) or idazoxan (IDA, α2-adrenergic receptor antagonist) on the bvPLA_2_-induced anti-allodynic action. As shown in Figure 4, IDA (1 mg/kg, i.p.) significantly blocked the relieving effect of bvPLA_2_ on oxaliplatin-induced cold and mechanical allodynia. However, PRA (10 mg/kg, i.p.) did not affect the bvPLA_2_ effect, because there were no significant differences in cold and mechanical sensitivity between the Oxa + PLA_2_ + NS and Oxa + PLA_2_ + PRA groups (*p* > 0.05, Table 1). These results indicate that bvPLA_2_ treatment alleviates oxaliplatin-induced acute cold and mechanical allodynia in mice via activation of α2-adrenergic receptors, but not α1-adrenergic receptors.

**Table 1:** Effect of α1-adrenergic receptor antagonist, prazosin (PRA) on bvPLA_2_-induced anti-allodynic action in oxaliplatin-administered mice.

Post-Oxaliplatin Day	D0	D3	D5	D7
Acetone test		Frequency		
Oxa + PBS + PRA/NS	2.1 ± 0.04	4.4 ± 0.08	3.6 ± 0.15	3.3 ± 0.09
Oxa + PLA_2_ + NS	2.0 ± 0.06	3.5 ± 0.15 ***	3.0 ± 0.10 **	2.6 ± 0.10 ***
Oxa + PLA_2_ + PRA	2.1 ± 0.06	3.1 ± 0.12 ***	3.1 ± 0.09 *	2.8 ± 0.07 **
von Frey test <0.4 g>		% withdrawal	response	
Oxa + PBS + PRA/NS	40.8 ± 0.83	76.7 ± 1.67	55.8 ± 1.54	44.2 ± 1.54
Oxa + PLA_2_ + NS	40.0 ± 1.83	59.2 ± 2.39 ***	48.1 ± 1.29 **	41.5 ± 1.12
Oxa + PLA_2_ + PRA	40.8 ± 2.01	57.5 ± 1.71 ***	47.5 ± 1.71 **	40.33 ± 1.67

Oxa + PBS + PRA/NS, *n* = 12; Other groups, *n* = 6 mice/group; Results are expressed as mean ± SEM; * *p* < 0.05, ** p < 0.01, *** p < 0.001, *vs.* Oxa + PBS + PRA/NS, one-way ANOVA followed by the Tukey’s multiple comparison test.

## 3. Discussion

Oxaliplatin-induced neuropathic pain represents a major obstacle to successful cancer treatment as it restricts both individual and cumulative dosages. However, despite these limitations, it is widely used and many patients suffer from the development of long-lasting consequences (*i.e.*, peripheral neuropathy) [28,29]. Patients may also experience cold-induced paresthesias, throat and jaw tightness, and occasionally focal weakness [30]. Oxaliplatin is structurally similar to other platinum based chemotherapy drugs, such as cisplatin and carboplatin. They all have neurotoxicity, however oxaliplatin has little nephrotoxicity and hematotoxicity [31]. It has been shown that oxaliplatin-induced acute neuropathy is characterized by a specific somatosensory profile, *i.e.*, cold and mechanical hypersensitivity [32]. Therefore, cold and mechanical hypersensitivity is a hallmark of oxaliplatin-induced neuropathy. Indeed, in this study, a single intraperitoneal administration of oxaliplatin (6 mg/kg) significantly increased the cold and mechanical sensitivity in mice, corroborating the previous reports using rats [33,34]. This mouse model might help in exploring the molecular and genetic mechanism of oxaliplatin-induced neuropathic pain in the future studies, since transgenic and gene knock-out/knock-in animals have been developed primarily in the mouse [35,36,37].

There have been few reports about the effective treatment and prevention of oxaliplatin-induced neuropathic pain. However, our previous study has shown that BV has a beneficial role in reliving oxaliplatin-induced neuropathic pain symptoms in rats, suggesting that BV could be an alternative therapeutic option for the management of oxaliplatin-induced peripheral neuropathy [9,16]. Interestingly, we recently found that bvPLA_2_ mitigates cisplatin-induced nephrotoxicity and acetaminophen-induced hepatotoxicity, which was more potent than BV [24,38]. In the present study, we investigated for the first time whether bvPLA_2_ has an analgesic effect on oxaliplain-induced cold and mechanical allodynia. Our data showed that the treatment of bvPLA_2_ significantly alleviated the allodynia in oxaliplatin-administered mice and such bvPLA_2_ effect was superior to the BV effect. This powerful analgesic effect of bvPLA_2_ led us to investigate the underlying mechanisms.

The extensive data support a role for the monoamine neurotransmitters (*i.e.*, serotonin and noradrenaline) and opioids, in the modulation of pain [39,40]. Serotonin and noradrenaline have been implicated as principal mediators of endogenous analgesic mechanisms in the descending pain pathways [39]. Our previous study suggested that the anti-allodynic effect of BV on oxaliplatin-induced cold allodynia in rats involves the noradrenergic, but not opioid, system [9]. In addition, spinal 5-HT_3_ receptors play an important role in the BV-induced anti-allodynic action in oxaliplatin-injected rats [16]. In contrast, the present study clearly showed that serotonin depletion by pretreatment of PCPA did not significantly affect the anti-allodynic effects of bvPLA_2_ in oxaliplatin-administered mice. This result suggests that the serotonergic inhibitory pathway is not involved in the analgesic effect of bvPLA_2_ on oxaliplatin-induced cold and mechanical hypersensitivity, unlike the case of BV. Other active components of BV, such as apamin [41], might be responsible for the serotonergic mechanism of BV-induced analgesia. However, in noradrenaline-depleted mice, the suppressive effect of bvPLA_2_ on oxaliplatin-induced cold and mechanical allodynia was significantly prevented. These results indicate that the noradrenergic analgesic system is at least partially involved in the analgesic effects of bvPLA_2_ in oxaliplatin-administered mice.

α-adrenergic receptors have been demonstrated to play an important role in the modulation of cold allodynia via the noradrenergic pain inhibitory system [42]. Previous studies showed that either α1- [43,44] or α2-adrenergic receptors [45,46] are responsible for the adrenergic sensitivity of nerve injury-induced neuropathic rats. A dual contribution of α1- and α2-adrenergic receptors to neuropathic pain was also suggested [47,48]. Also other articles have elucidated that the analgesic effects of BV are mediated mainly by activation of α2-adrenergic receptors in various pain models, such as nerve injury-induced neuropathic pain, acetic acid-induced visceral pain and inflammatory pain [12,13,14,15]. Our recent study also showed that α2-adrenergic receptors mediate the anti-allodynic effect of BV in oxaliplatin-induced neuropathic pain in rats [9]. In the present study, we further examined which adrenergic receptor subtype mediates the effects of bvPLA_2_ on cold and mechanical allodynia in oxaliplatin-administered mice. The current results showed that IDA (α2-adrenergic antagonist) was able to completely block the anti-allodynic effect of bvPLA_2_ on oxaliplatin-induced neuropathic pain in mice, whereas PRA (α1-adrenergic antagonist) did not. These results indicate that the noradrenergic mechanism of the analgesic effect of bvPLA_2_ on oxaliplatin-induced neuropathic pain is mediated by activation of α2-adrenergic, but not α1-adrenergic, receptors.

In this study, we have clearly shown that bvPLA_2_ treatment strongly alleviates oxaliplatin-induced acute cold and mechanical allodynia in mice through the activation of the noradrenergic system, via α2-adrenegic receptors. Besides such action through the neurochemical mechanism, bvPLA_2_ is known to have a potent immune modulatory effect. The major pathway of the bvPLA_2_-induced immune modulation is to increase peripheral regulatory T cells, which play a key role in the maintenance of tolerance in the immune system. Our recent studies showed that such strategies using bvPLA_2_ could be successful in the prevention of cisplatin-induced acute kidney and acetaminophen-induced acute liver injury, by suppressing immune response via the modulation of regulatory T cells [24,38]. Another previous study also demonstrated that regulatory T cells attenuate peripheral nerve injury-induced neuropathic pain in rats [49]. Thus, it would be of high interest to see if bvPLA_2_ treatment before an oxaliplatin administration prevents the development of neuropathic cold and mechanical allodynia by regulating peripheral immune response. To our best knowledge, there are no clinical trials for bvPLA_2_ treatment. Further research is required in this field. In addition, our current work is limited to behavioral and pharmacological approaches. Molecular and genetic studies using the advantage of the mouse model are now in progress to elucidate more detailed mechanism of bvPLA_2_-induced analgesia.

## 4. Experimental Section 

### 4.1. Animals

Male C57BL/6 mice (6–8 weeks old) were purchased from the Daehan Biolink (Chungbuk, Korea). They were kept under specific pathogen-free conditions with air conditioning and a 12 h light/dark cycle. The mice had free access to food and water during the experiments. The study was approved by the Kyung Hee University Animal Care and Use Committee (KHUASP(SE)-15-024).

### 4.2. Behavioral Tests

Behavioral tests representing different sensory components of neuropathic pain were conducted before and after an oxaliplatin administration. The mice were habituated to handling by investigators and to all testing procedures for a week before the start of the experiments. The experimenters were blind to oxaliplatin and any treatments.

Cold sensitivity was measured by an acetone test [50]. Mice were placed in a clear plastic box (12 × 8 × 6 cm) with a wire mesh floor and allowed to habituate for 30 min prior to the testing. Acetone (10 μL, Reagents Chemical Ltd., Gyonggi-do, Korea) was sprayed onto the plantar skin of each hind paw 3 times and the frequency of licking and shaking of the affected paw was counted after the acetone spray for 30 s. The advantage of acetone test is that it is quite simple, economical, and that the assessment for multiple mice can be made in a short period of time. The disadvantage is that acetone can also induce some behavioral response in naïve mice. However, mice having allodynia (e.g., oxaliplatin-administered mice) show a significant increase in the level of response to acetone when compared to the control mice.

Mechanical sensitivity was measured by the von Frey hair test [51]. Mice were placed in a clear plastic box (12 × 8 × 6 cm) with a wire mesh floor and allowed to habituate for 30 min before the testing. A von Frey filament (Linton Instrumentation, Norfolk, UK) with a bending force of 0.4 g were applied to the midplantar skin (avoiding the base of the tori) of each hind paw 10 times, with each application held for 3 s [52]. The number of withdrawal responses to the von Frey filament applications from both hind paws were counted and then expressed as an overall percentage response.

### 4.3. Oxaliplatin Administration and BV/bvPLA_2_ Treatment

Oxaliplatin (Sigma, St. Louis, MO, USA) was dissolved in a 5% glucose solution at a concentration of 2 mg/mL and was intraperitoneally (i.p.) injected at 6 mg/kg [9,33]. The vehicle control group received the same volume of a 5% glucose solution through the same injection route.

BV (1 mg/kg, i.p., Sigma) or bvPLA_2_ (0.2 mg/kg, i.p., Sigma) [9,24,38] dissolved in phosphate buffered saline (PBS) was injected in oxaliplatin-administered mice for a continuous five days. Cold and mechanical sensitivity were measured by acetone test and von Frey hair test, respectively, before each bvPLA_2_ or BV treatment. Control group was treated intraperitoneally with PBS.

### 4.4. Depletion of Serotonin or Noradrenaline

DL-*p*-chlorophenylalanine (PCPA, Sigma, an inhibitor of serotonin synthesis, 150 mg/kg/day) or vehicle (normal saline, NS) was injected intraperitoneally to mice prior to oxaliplatin administration for three days. The dosage and treatment course of PCPA have been widely used to deplete 5-HT stores [25,26]. After 5-HT depletion with PCPA, oxaliplatin and bvPLA2 were administered as aforementioned.

*N*-(2-Chloroethyl)-*N*-ethyl-2-bromobenzylamine hydrochloride (DSP4, TOCRIS, 50 mg/kg) or vehicle (NS) was injected intraperitoneally to mice a day before an oxaliplatin administration. DSP4 at a concentration of 50 mg/kg has been shown to be an effective dose for maximal NE depletion [53], with the advantage that mice did not require special care following injection as no adverse effects could be observed.

### 4.5. α1- or α2-Adrenergic Receptor Antagonist

To test which adrenergic receptor subtype mediates the anti-allodynic effects of bvPLA_2_ in oxaliplatin-administered mice, specific antagonists were administered intraperitoneally 30 min prior to bvPLA_2_ treatement for five days: α1-adrenergic receptor antagonist (prazosin, 10 mg/kg, Sigma), α2-adrenergic receptor antagonist (idazoxan, 1 mg/kg, Sigma). The dose of each antagonist was determined based on the previous studies showing the selective and effective antagonistic action against adrenergic receptor-mediated responses [54,55,56].

### 4.6. Statistical Analyses

The data are presented as mean ± SEM and were analyzed by the unpaired t-test or one-way ANOVA followed by the Tukey’s multiple comparison test to determine the statistical differences among the groups*.* *p* < 0.05 was considered as statistically significant.

## 5. Conclusions

In conclusion, our findings reveal that cold and mechanical sensitivity were significantly increased after a single injection of oxaliplatin in mice. BV and bvPLA_2_ can exert significant relieving effects on oxaliplatin-induced cold and mechanical hypersensitivity, in which bvPLA_2_ is more potent than BV. The serotonergic mechanism is not involved in the analgesic effect of bvPLA_2_ on oxaliplatin-induced neuropathic pain, whereas the noradrenergic pain inhibitory system at least partially mediates the bvPLA_2_ effect. Finally, we demonstrated that bvPLA_2_ treatment alleviates oxaliplatin-induced acute cold and mechanical allodynia in mice via activation of α2-adrenergic receptors. These findings may provide clinically useful evidence for the application of bvPLA_2_ in the management of peripheral neuropathic pain that occurs after the oxaliplatin administration.

## References

[1] Andre T., Boni C., Mounedji-Boudiaf L., Navarro M., Tabernero J., Hickish T., Topham C., Zaninelli M., Clingan P., Bridgewater J. (2004). Oxaliplatin, fluorouracil, and leucovorin as adjuvant treatment for colon cancer. N. Engl. J. Med..

[2] De Gramont A., Figer A., Seymour M., Homerin M., Hmissi A., Cassidy J., Boni C., Cortes-Funes H., Cervantes A., Freyer G. (2000). Leucovorin and fluorouracil with or without oxaliplatin as first-line treatment in advanced colorectal cancer. J. Clin. Oncol..

[3] Cassidy J., Misset J.L. (2002). Oxaliplatin-related side effects: Characteristics and management. Semin. Oncol..

[4] Extra J.M., Marty M., Brienza S., Misset J.L. (1998). Pharmacokinetics and safety profile of oxaliplatin. Semin. Oncol..

[5] Pasetto L.M., D’Andrea M.R., Rossi E., Monfardini S. (2006). Oxaliplatin-related neurotoxicity: How and why?. Crit. Rev. Oncol. Hematol..

[6] Grothey A. (2003). Oxaliplatin-safety profile: Neurotoxicity. Semin. Oncol..

[7] Billingham M.E., Morley J., Hanson J.M., Shipolini R.A., Vernon C.A. (1973). Letter: An anti-inflammatory peptide from bee venom. Nature.

[8] Choi M.S., Park S., Choi T., Lee G., Haam K.K., Hong M.C., Min B.I., Bae H. (2013). Bee venom ameliorates ovalbumin induced allergic asthma via modulating CD4+CD25+ regulatory T cells in mice. Cytokine.

[9] Lim B.S., Moon H.J., Li D.X., Gil M., Min J.K., Lee G., Bae H., Kim S.K., Min B.I. (2013). Effect of bee venom acupuncture on oxaliplatin-induced cold allodynia in rats. Evid. Based Complement. Alternat. Med..

[10] Yoon S.Y., Yeo J.H., Han S.D., Bong D.J., Oh B., Roh D.H. (2013). Diluted bee venom injection reduces ipsilateral mechanical allodynia in oxaliplatin-induced neuropathic mice. Biol. Pharm. Bull..

[11] Somerfield S.D., Brandwein S. (1988). Bee venom and adjuvant arthritis. J. Rheumatol..

[12] Roh D.H., Kwon Y.B., Kim H.W., Ham T.W., Yoon S.Y., Kang S.Y., Han H.J., Lee H.J., Beitz A.J., Lee J.H. (2004). Acupoint stimulation with diluted bee venom (apipuncture) alleviates thermal hyperalgesia in a rodent neuropathic pain model: Involvement of spinal alpha 2-adrenoceptors. J. Pain.

[13] Baek Y.H., Huh J.E., Lee J.D., Choi do Y., Park D.S. (2006). Antinociceptive effect and the mechanism of bee venom acupuncture (apipuncture) on inflammatory pain in the rat model of collagen-induced arthritis: Mediation by alpha2-adrenoceptors. Brain Res..

[14] Kwon Y.B., Kang M.S., Han H.J., Beitz A.J., Lee J.H. (2001). Visceral antinociception produced by bee venom stimulation of the Zhongwan acupuncture point in mice: Role of alpha(2) adrenoceptors. Neurosci. Lett..

[15] Kim H.W., Kwon Y.B., Han H.J., Yang I.S., Beitz A.J., Lee J.H. (2005). Antinociceptive mechanisms associated with diluted bee venom acupuncture (apipuncture) in the rat formalin test: Involvement of descending adrenergic and serotonergic pathways. Pharmacol. Res..

[16] Lee J.H., Li D.X., Yoon H., Go D., Quan F.S., Min B.I., Kim S.K. (2014). Serotonergic mechanism of the relieving effect of bee venom acupuncture on oxaliplatin-induced neuropathic cold allodynia in rats. BMC Complement. Altern. Med..

[17] Kang S.Y., Roh D.H., Park J.H., Lee H.J., Lee J.H. (2012). Activation of spinal alpha2-adrenoceptors using diluted bee venom stimulation reduces cold allodynia in neuropathic pain rats. Evid. Based Complement. Altern. Med..

[18] Kang S.Y., Roh D.H., Yoon S.Y., Moon J.Y., Kim H.W., Lee H.J., Beitz A.J., Lee J.H. (2012). Repetitive treatment with diluted bee venom reduces neuropathic pain via potentiation of locus coeruleus noradrenergic neuronal activity and modulation of spinal NR1 phosphorylation in rats. J. Pain.

[19] Monti M.C., Casapullo A., Santomauro C., D’Auria M.V., Riccio R., Gomez-Paloma L. (2006). The molecular mechanism of bee venom phospholipase A2 inactivation by bolinaquinone. Chembiochem.

[20] Zhao H., Kinnunen P.K. (2003). Modulation of the activity of secretory phospholipase A2 by antimicrobial peptides. Antimicrob. Agents Chemother..

[21] Jeong J.K., Moon M.H., Bae B.C., Lee Y.J., Seol J.W., Park S.Y. (2011). Bee venom phospholipase A2 prevents prion peptide induced-cell death in neuronal cells. Int. J. Mol. Med..

[22] Lopez-Vales R., Ghasemlou N., Redensek A., Kerr B.J., Barbayianni E., Antonopoulou G., Baskakis C., Rathore K.I., Constantinou-Kokotou V., Stephens D. (2011). Phospholipase A2 superfamily members play divergent roles after spinal cord injury. FASEB J..

[23] Burrage P.S., Mix K.S., Brinckerhoff C.E. (2006). Matrix metalloproteinases: Role in arthritis. Front. Biosci..

[24] Kim H., Lee H., Lee G., Jang H., Kim S.-S., Yoon H., Kang G.-H., Hwang D.-S., Kim S.K., Chung H.-S. (2015). Phospholipase A2 inhibits cisplatin-induced acute kidney injury by modulating regulatory T cells via CD206 mannose receptor. Kidney Int..

[25] Zhu J.X., Zhu X.Y., Owyang C., Li Y. (2001). Intestinal serotonin acts as a paracrine substance to mediate vagal signal transmission evoked by luminal factors in the rat. J. Physiol..

[26] Maleki N., Nayebi A.M., Garjani A. (2005). Effects of central and peripheral depletion of serotonergic system on carrageenan-induced paw oedema. Int. Immunopharmacol..

[27] Kudo T., Kushikata T., Kudo M., Hirota K. (2011). Antinociceptive effects of neurotropin in a rat model of central neuropathic pain: DSP-4 induced noradrenergic lesion. Neurosci. Lett..

[28] Farquhar-Smith P. (2011). Chemotherapy-induced neuropathic pain. Curr. Opin. Support. Palliat. Care.

[29] Kannarkat G., Lasher E.E., Schiff D. (2007). Neurologic complications of chemotherapy agents. Curr. Opin. Neurol..

[30] Lehky T.J., Leonard G.D., Wilson R.H., Grem J.L., Floeter M.K. (2004). Oxaliplatin-induced neurotoxicity: Acute hyperexcitability and chronic neuropathy. Muscle Nerve.

[31] Desoize B., Madoulet C. (2002). Particular aspects of platinum compounds used at present in cancer treatment. Crit. Rev. Oncol. Hematol..

[32] Binder A., Stengel M., Maag R., Wasner G., Schoch R., Moosig F., Schommer B., Baron R. (2007). Pain in oxaliplatin-induced neuropathy––Sensitisation in the peripheral and central nociceptive system. Eur. J. Cancer.

[33] Ling B., Coudore-Civiale M.A., Balayssac D., Eschalier A., Coudore F., Authier N. (2007). Behavioral and immunohistological assessment of painful neuropathy induced by a single oxaliplatin injection in the rat. Toxicology.

[34] Ling B., Coudore F., Decalonne L., Eschalier A., Authier N. (2008). Comparative antiallodynic activity of morphine, pregabalin and lidocaine in a rat model of neuropathic pain produced by one oxaliplatin injection. Neuropharmacology.

[35] De Felipe C., Herrero J.F., O’Brien J.A., Palmer J.A., Doyle C.A., Smith A.J., Laird J.M., Belmonte C., Cervero F., Hunt S.P. (1998). Altered nociception, analgesia and aggression in mice lacking the receptor for substance P. Nature.

[36] Honore P., Rogers S.D., Schwei M.J., Salak-Johnson J.L., Luger N.M., Sabino M.C., Clohisy D.R., Mantyh P.W. (2000). Murine models of inflammatory, neuropathic and cancer pain each generates a unique set of neurochemical changes in the spinal cord and sensory neurons. Neuroscience.

[37] Back S.K., Sung B., Hong S.K., Na H.S. (2002). A mouse model for peripheral neuropathy produced by a partial injury of the nerve supplying the tail. Neurosci. Lett..

[38] Kim H., Keum D.J., Kwak J., Chung H.S., Bae H. (2014). Bee venom phospholipase A2 protects against acetaminophen-induced acute liver injury by modulating regulatory T cells and IL-10 in mice. PLoS ONE.

[39] Lamont L.A., Tranquilli W.J., Grimm K.A. (2000). Physiology of pain. Vet. Clin. N. Am. Small Anim. Pract..

[40] Ossipov M.H., Morimura K., Porreca F. (2014). Descending pain modulation and chronification of pain. Curr. Opin. Support. Palliat. Care.

[41] Crespi F. (2009). Apamin increases 5-HT cell firing in raphe dorsalis and extracellular 5-HT levels in amygdala: A concomitant *in vivo* study in anesthetized rats. Brain Res..

[42] Millan M.J. (2002). Descending control of pain. Prog. Neurobiol..

[43] Korenman E.M., Devor M. (1981). Ectopic adrenergic sensitivity in damaged peripheral nerve axons in the rat. Exp. Neurol..

[44] Lee D.H., Liu X., Kim H.T., Chung K., Chung J.M. (1999). Receptor subtype mediating the adrenergic sensitivity of pain behavior and ectopic discharges in neuropathic Lewis rats. J. Neurophysiol..

[45] Leem J.W., Gwak Y.S., Nam T.S., Paik K.S. (1997). Involvement of alpha2-adrenoceptors in mediating sympathetic excitation of injured dorsal root ganglion neurons in rats with spinal nerve ligation. Neurosci. Lett..

[46] Zhang J.M., Song X.J., LaMotte R.H. (1997). An *in vitro* study of ectopic discharge generation and adrenergic sensitivity in the intact, nerve-injured rat dorsal root ganglion. Pain.

[47] Hord A.H., Denson D.D., Stowe B., Haygood R.M. (2001). Alpha-1 and alpha-2 adrenergic antagonists relieve thermal hyperalgesia in experimental mononeuropathy from chronic constriction injury. Anesth. Analg..

[48] Tracey D.J., Cunningham J.E., Romm M.A. (1995). Peripheral hyperalgesia in experimental neuropathy: Mediation by alpha 2-adrenoreceptors on post-ganglionic sympathetic terminals. Pain.

[49] Austin P.J., Kim C.F., Perera C.J., Moalem-Taylor G. (2012). Regulatory T cells attenuate neuropathic pain following peripheral nerve injury and experimental autoimmune neuritis. Pain.

[50] Flatters S.J., Bennett G.J. (2004). Ethosuximide reverses paclitaxel- and vincristine-induced painful peripheral neuropathy. Pain.

[51] Joseph E.K., Levine J.D. (2009). Comparison of oxaliplatin- and cisplatin-induced painful peripheral neuropathy in the rat. J. Pain.

[52] Shibata K., Sugawara T., Fujishita K., Shinozaki Y., Matsukawa T., Suzuki T., Koizumi S. (2011). The astrocyte-targeted therapy by bushi for the neuropathic pain in mice. PLoS ONE.

[53] Scullion G.A., Kendall D.A., Sunter D., Marsden C.A., Pardon M.C. (2009). Central noradrenergic depletion by DSP-4 prevents stress-induced memory impairments in the object recognition task. Neuroscience.

[54] Johnson J.D., Campisi J., Sharkey C.M., Kennedy S.L., Nickerson M., Greenwood B.N., Fleshner M. (2005). Catecholamines mediate stress-induced increases in peripheral and central inflammatory cytokines. Neuroscience.

[55] Nelson L.E., Lu J., Guo T., Saper C.B., Franks N.P., Maze M. (2003). The alpha2-adrenoceptor agonist dexmedetomidine converges on an endogenous sleep-promoting pathway to exert its sedative effects. Anesthesiology.

[56] Zarrindast M.R., Homayoun H., Khavandgar S., Fayaz-Dastgerdi M. (2002). The effects of simultaneous administration of alpha(2)-adrenergic agents with L-NAME or L-arginine on the development and expression of morphine dependence in mice. Behav. Pharmacol..

